# A novel refined pyroptosis and inflammasome-related genes signature for predicting prognosis and immune microenvironment in pancreatic ductal adenocarcinoma

**DOI:** 10.1038/s41598-022-22864-z

**Published:** 2022-11-01

**Authors:** Jieliang Zuo, Chenhe Yi, Zhenmei Chen, Bo Zhou, Tingsong Yang, Jing Lin

**Affiliations:** 1 Department of General Surgery, Shanghai Tenth People’s Hospital, Tongji University School of Medicine, Shanghai, 200072 People’s Republic of China; 2 Department of General Surgery, Huashan Hospital, Fudan University, 12 Middle Wulumuqi Road, Shanghai, 200040 People’s Republic of China; 3 Cancer Metastasis Institute, Fudan University, Shanghai, 200040 People’s Republic of China

**Keywords:** Pancreatic cancer, Computational biology and bioinformatics

## Abstract

Pyroptosis is an inflammatory form of cell death, which plays a key role in the development of auto-inflammation and cancer. This study aimed to construct a pyroptosis and inflammasome-related genes for predicting prognosis of the pancreatic ductal adenocarcinoma (PDAC). This study was based primarily on the one-way analysis of variance, univariate Cox regression analysis, Least absolute shrinkage and selection operator (LASSO) Cox regression, a risk-prognostic signature, gene set variation analysis (GSVA), and immune microenvironment analysis, using PDAC data from The Cancer Genome Atlas and International Cancer Genome Consortium databases for the analysis of the role of 676 pyroptosis and inflammasome-related genes in PDAC retrieved from the Reactome and GeneCards databases. Lastly, we collected six paired PDAC and matched normal adjacent tissue samples to verify the expression of signature genes by quantitative real-time PCR (qRT-PCR). We identified 18 candidate pyroptosis and inflammasome-related genes that differed significantly between pathologic grades (stages) of PDAC patients. The univariate Cox and LASSO analyses pointed to six genes as the best variables for constructing a prognostic signature, including *ACTA2*, *C1QTNF9*, *DNAH8*, *GATM*, *LBP*, and *NGF*. The results of the risk prognostic model indicated that the AUCs at 1, 3, and 5 years were greater than 0.62. GSVA revealed that ‘GLYCOLYSIS’, ‘P53 PATHWAY’, ‘KRAS SIGNALING UP’, and ‘INFLAMMATORY RESPONSE’ hallmark gene sets were associated with the risk score. The high-risk group was associated with poor prognosis and was characterized by a lower infiltration of cells involved in anti-tumor immunity; whereas the low-risk group with higher T cells, NK cells, and macrophages showed relatively better survival and significantly higher upregulation of cytolytic scores and inflammation scores. Additionally, crucial pyroptosis and inflammasome-related genes were further validated by qRT-PCR. Our study revealed the prognostic role of the pyroptosis and inflammasome-related genes in PDAC for the first time. Simultaneously, the biological and prognostic heterogeneity of PDAC had been demonstrated, deepening our molecular understanding of this tumor.

## Introduction

Pancreatic ductal adenocarcinoma (PDAC) accounts for more than 90% of pancreatic tumors, with a five-year survival of less than 5%^1–3^. At present, the optimal treatment for patients with PDAC is surgical resection. However, PDAC patients are often diagnosed with non-resectable metastasis^4,5^. Moreover, operative mortality has significantly declined to less than 5% in the last decade, but the incidence of postoperative morbidity remains high at 40% to 50%^6,7^. Therefore, exploration of molecular biomarkers and predicting survival is paramount for the precision treatment of PDAC patients.

Pyroptosis is a form of inflammatory programmed cell death with the synthesis and release of a large number of inflammatory factors such as IL-1b (Interleukin-1b) and IL-18 (Interleukin-18)^8^. Pyroptosis was first discovered in the defense of pathogenic insults and later was found that play a critical role in many inflammatory diseases^9^. The canonical pathway of pyroptosis is mostly induced by the inflammasome, including NLRP1, NLRP3, NLRC4, AIM2, and the pyrin domain^10^. These inflammasomes recruit the apoptosis-related dot-like protein ASC and pro-caspase-1 to form the inflammasome complex^11^. Caspase-1 has lately been activated to cleave GSDMD to produce GSDMD-NT (N-terminal) which will form oligomers to play the function of drilling and induce pyroptosis^12^.

In terms of cancer, pyroptosis might play dual roles in terms of tumorigenesis. Pyroptosis-promotor GSDMD-knockout could inhibit the growth of non-small cell lung cancer (NSCLC) cells^13^. On the contrary, GSDMD-silence promoted the development of gastric cancer^14^. Similarly, the functions of the inflammasome in the tumor are also complicated. Activation of the inflammasome-related gene of NLRP3 suppressed the development of hepatocellular carcinoma cells^15^. Nonetheless, NLRP3 promoted migration and invasion of colorectal cancer cells by regulating Snail1 expression^16^. In PDAC, few studies have focused on pyroptosis. Mammalian STE20-like kinase 1 (MST1) was reported to promote PDAC cell death and inhibit the proliferation, migration, and invasion of PDAC cells by inducing caspase-1-mediated pyroptosis^17^. However, the relationship between PDAC and pyroptosis remains unclear. In addition, inflammasome is widely expressed in immune cells. It is unclear whether aberrantly inflammasome signaling in the tumor microenvironment could thwarts immune surveillance and promotes PDAC development^18^. Therefore, the role of pyroptosis and inflammasome-related genes in PDAC needs to be further explored.

In this study, pyroptosis and inflammasome-associated differentially-expressed genes were screened from the TCGA database and a new risk score model was developed to predict the prognosis of patients with PDAC. The specific Prognostic Scoring System of PDAC were validated to stratify patients into different survival and immune function, which might guide therapeutic strategies of PDAC in the future.The results were further verified in clinical specimens from Shanghai Tenth People's Hospital (SHDSYY) through quantitative real-time PCR (qRT-PCR) .

## Methods

### Data source

The datasets supporting the conclusions of this article are available in The Cancer Genome Atlas (TCGA) [https://portal.gdc.cancer.gov/] and International Cancer Genome Consortium (ICGC) [https://dcc.icgc.org/] repository.

145 PDAC samples were selected as the training set in TCGA database (Supplementary Table 1) after excluding samples with incomplete Pathologic Grade and overall survival (OS) records, mainly for the gene screening, construction, and assessment of the prognostic signature, functional enrichment, and immune landscape analysis. The 177 PDAC samples from the ICGC database (Supplementary Table 2) containing detailed survival information were used in this study primarily as a validation set to examine the prevalence of the prognostic signature.

### Access to the pyroptosis and inflammasome-related genes

Using the keywords ‘pyroptosis’ and ‘inflammasome’, 27 pyroptosis-associated genes and 21 inflammasome-associated genes were retrieved from the Reactome database; 120 Pyroptosis- and 639 Inflammasome-associated genes were obtained from the GeneCards database. The remaining 676 genes after de-duplication were considered pyroptosis and inflammasome-related genes (Table 1). Subsequently, candidate pyroptosis and inflammasome-related genes were obtained by removing genes that were not expressed in more than 50% of the samples, containing 654 candidate genes (Supplementary Table 3).

**Table 1:** pyroptosis and inflammasome-related genes.

Dataset	Gene	Number of genes
REACTOME_PYROPTOSIS	AIM2, APP, BCL2, BCL2L1, CASP1, HMOX1, HSP90AB1, MEFV, NFKB1, NFKB2, NLRC4, NLRP1, NLRP3, P2RX7, PANX1, PSTPIP1, PYCARD, RELA, SUGT1, TXN, TXNIP	27
REACTOME_INFLAMMASOMES	BAK1, BAX, CASP1, CASP3, CASP4, CASP5, CHMP2A, CHMP2B, CHMP3, CHMP4A, CHMP4B, CHMP4C, CHMP6, CHMP7, CYCS, ELANE, GSDMD, GSDME, GZMB, HMGB1, IL18, IL1A, IL1B, IRF1, IRF2, TP53, TP63	21
GENECARD_PYROPTOSIS	GSDMD, GSDME, NLRP3, CASP1, CASP4, GSDMB, GSDMC, IL1B, GZMB, NLRP1, GSDMA, GZMA, NLRC4, CASP5, AIM2, PYCARD, CASP3, DHX9, NLRP9, NAIP, HMGB1, CASP8, FOXO3, IL18, APIP, TXNIP, GBP1, CASP6, NEK7, GJA1, P2RX7, TP53, MALT1, AGER, TET2, EEF2K, CD274, FGF21, CEBPB, TFAM, STK4, PRDM1, PRF1, MST1, ELAVL1, TREM2, HDAC6, SQSTM1, IRF3, STING1, ZBP1, PECAM1, DDX3X, PRTN3, SERPINB1, NR1H2, CAMP, MRE11, PARP1, CTSG, GBP5, NLRP7, MKI67, IL36G, IL36B, CPTP, BNIP3, ANO6, FADD, MEFV, APOL1, TNF, VIM, CAPN1, JUN, ALK, SIRT1, BIRC3, BIRC2, UBE2D2, LY96, RIPK3, GLMN, IRGM, NLRP13, TUBB6, NOS2, NOS1, PYDC2, IFI16, AKT1, EGFR, TP63, ATF6, IRF1, IRF2, POP1, ORMDL3, MDM2, BTK, NFKB1, STAT3, BCL2, TLR2, ANXA2, IL1RN, BECN1, CD14, GSTO1, IL13, CHI3L1, PANX1, LRPPRC, CXCL8, IL13RA2, IL32, BST2, GPER1, LYST, CLEC5A	120
GENECARD_INFLAMMASOMES	NLRP3, NLRP1, IL1B, PYCARD, CASP1, AIM2, NLRC4, MEFV, CASP4, CARD8, IL18, IFI16, GSDMD, NLRP9, NLRP6, P2RX7, HMGB1, TLR4, NAIP, DDX3X, TLR2, DHX33, SYK, PSTPIP1, GBP5, CASP5, PYDC2, NLRP2, CASP8, EIF2AK2, TXNIP, NLRC3, NLRP7, PYDC5, NLRP12, NOD2, NLRX1, RELA, IL1RN, SQSTM1, CGAS, IL1A, RIPK3, PANX1, CPTP, NEK7, CD36, DHX9, NFKB1, LRRFIP2, WDR1, BRCC3, APP, TNFAIP3, TLR8, TNF, SREBF2, CLEC7A, ARRB2, FFAR1, FFAR4, MYD88, IL6, STMP1, CTSB, SNCA, NOD1, AGER, LACC1, NFE2L2, SIRT1, NLRP13, BTK, MAPK13, PML, C5, IL1R1, NLRP14, SREBF1, BCL2, TLR6, DDX58, MAVS, CNR1, TLR9, STING1, CASP6, MAPK1, MAP1LC3B, LGALS3, HMOX1, CYBB, PYDC1, MAP1LC3A, HK1, BECN1, MAPRE1, FOXO3, IL1RAPL2, IL10, CAMP, UCP2, DDX19A, RBX1, ZBP1, BIRC3, CASR, ITGB1, ITGA5, APOE, TLR7, NLRC5, STAMBP, TRAF2, IL23A, OPTN, P2RX4, POP1, BRCA1, IL22, NLRP10, CASP7, MAPK14, USP50, WAS, IL18BP, ATG16L1, CEBPB, JUN, CFHR1, OLR1, MAPK8, MALT1, NOX1, SAA1, IKBKE, MIF, IL17A, TRAF6, FASN, CARD18, GSDME, FSTL1, MTOR, FADD, MLKL, TIFA, TRIM31, RNASEL, CXCL8, RIPK1, GLTP, IRF7, CFH, TRIM25, FLT3, TRPM2, PPARG, NR1H3, NRG1, DPP9, ILF2, SUGT1, IFNG, PGM1, USP7, STUB1, CUL1, SKP1, FLII, ATP1B3, UBR5, USP47, MARCHF7, TLR3, GJA1, DICER1, CD14, TSPO, DPEP1, CYLD, AGT, FLNA, HIF1A, PLK4, CRKL, TAB2, EIF4G1, SUMO1, ADIPOQ, RALB, NUP214, HAVCR2, EIF4G2, EIF4B, ERC1, RANGAP1, SNX5, ALMS1, HNRNPC, MAP4, IAPP, PDLIM1, NEDD8, EPS15L1, CC2D1A, AMOT, GIGYF2, SENP3, PDLIM5, PDLIM7, CIAPIN1, SNX2, AMOTL1, DCP1A, ARIH2, CLINT1, LARP1, NSFL1C, CORO1B, YEATS2, GEMIN5, SEPTIN9, SERBP1, TNRC6B, C1QTNF9, SKA3, TNKS1BP1, CCDC85C, RPAP3, SPATA2, PRRC2C, SPATA2L, MRTFB, A3GALT2, H2BU1, BCL2L1, NLRP11, CHEK2, PTPN11, SLC25A4, SIRT3, DNM1L, P2RY2, BIRC2, UBE2D2, HSF2, APOC3, GLMN, DDX10, H2AX, WDR90, STAT1, ZFP36, IL27, ALK, HDAC6, ESR2, PTGDR, P2RY1, UBE2D3, UBE2N, TRIM33, HCRT, SCAP, UBE2E1, S100A12, MUC5AC, TSLP, CDK5, JAK2, ADAM10, MAP3K7, LDLR, RAC1, KEAP1, FYN, PTGS2, TRAF3, EDN1, CUL3, BCL10, MARK4, CARD9, GSTO1, FCER1G, NR0B2, NFAT5, FCGR1A, DROSHA, GPRC6A, UBE2O, UBR2, TRIM11, CARD6, IRGM, HCAR1, ZNF7, GPSM3, DYSF, CEACAM1, XDH, NLRP4, ITGB2, CCL2, MUC1, ITGAM, PTPN2, CNR2, PKM, UBE2D1, DUSP10, SYNGAP1, UBE2G1, SOCS1, RAB1A, DEPTOR, FBXL2, CLEC5A, MT-CYB, NLRP8, AKT1, JAK1, NGF, FN1, BMPR2, CARD11, C3, ATF6, MCM4, ATF4, IL6ST, NR1I2, IL2, LIG3, ITPKC, RBP4, RHOB, SEMA4D, MYO1C, GZMA, NUP107, LEMD3, LTBR, MTDH, TMPRSS15, TPM4, NAA15, ACKR2, INPP4B, PLXNB2, TSHZ2, ARMC2, FSTL5, FUNDC1, FAM184A, CEP131, ZFP91, ZER1, DNAJC28, ZYG11B, DRC7, ABRAXAS2, CASP12, GABARAP, GABARAPL2, EGFR, MERTK, NR3C1, ACTA2, STIM1, NPPA, TFAM, PLIN2, TAGLN, S100A8, S100A9, UMOD, PHB2, ADGRE2, CFHR2, IRF3, ATN1, GBP1, ULK1, GABARAPL1, TRIM21, MAP1LC3C, IRAK3, CARD16, NLRP2B, GATM, TET2, VDR, HSP90AA1, CHRNA7, CNOT8, PLA2G6, DMD, NOX4, SNRNP70, PRKN, DCP2, HSPD1, LY96, SERPINA1, KPNA1, VEGFA, CARD17, NRXN3, FANCA, FANCC, CHI3L1, IL37, TXN, TLR5, MVK, MAP2K1, EP300, SESN2, TMBIM6, H2BC21, H2AC18, TIRAP, USP8, MLX, NPPB, CEBPE, TFEB, MID2, TRIM22, TRIM8, TRIM65, SOD2, PRKD1, ABCA1, TP73, CHRNA5, IL19, IL20, ARRDC3, MMP2, MMP9, PINK1, DDX6, S100B, BCL2L11, ANGPTL4, ERN1, MOG, CBLB, HSPA1A, HSPB8, IL12B, IL13, CD209, RNF31, RBCK1, IL13RA2, SHARPIN, MRE11, TOMM40, ELOVL6, CHUK, IL1RAP, JUNB, TICAM2, RIPK2, CD40, NCF1, CYBA, HSP90AB1, SIRT2, ATAT1, PTPN12, CRP, CARD14, LPIN2, IL1RL2, SAA4, SIGLEC5, FCHO2, PSTPIP2, IFIH1, HSPA4, NLRP5, TP53, IFNA1, PTPN22, TYK2, VIM, CAPN1, KCNN4, TGM2, PDCD1, APOA1, HPSE, PRF1, CAPN5, MKI67, KCNA3, SAMHD1, TREM2, TREX1, GSDMC, TGFB1, CASP3, CASP9, IRAK4, IRF1, HLA-G, IRF2, SLC22A12, CCL5, FAF1, PSMC5, CCL4, IL33, CLEC4D, SARM1, IKBKB, NFKB2, MAPK10, NFKBIA, FOS, MAP2K3, IKBKG, MAPK11, MAPK9, MAPK12, MAP2K6, TLR1, MAP2K4, MAP2K7, REL, RELB, FOSL1, TICAM1, NFKBIB, FOSB, JUND, NFKBIE, TLR10, SCNN1B, UBE3A, CD46, CANT1, ATG5, IL15, FNDC5, SLC2A1, IRAK1, ACE2, CFLAR, HMGCR, FURIN, BCL6, C7, ANO6, SLPI, DNAH8, ATM, PIK3CA, STAT3, SOD1, CFTR, CREB1, NOS2, TBK1, ANXA2, INS, PIK3CB, PIK3CG, NR1H2, ECE1, SLC6A4, FGF2, CD44, DUSP1, HSPA8, HTR1A, LEP, APAF1, HTR3A, OGG1, PKD2, TGIF1, TNFSF10, CTLA4, CCR7, CD274, INPP5D, HELLS, KLF4, EHMT1, ENPP2, C9, LBP, ATP6V0A2, SUV39H1, VASP, ERAP1, UBC, FOXG1, ACTR3, CDKN3, CXCL1, IL18R1, SRD5A2, CCL19, IFNB1, TUBB6, FLG, IL1F10, OSBP, RSAD2, ORMDL3, SIGLEC1, SPAST, CCL3, NAPRT, SLC30A6, CXCL9, ITLN1, MATN2, OSBPL1A, IL36G, ENTPD7, ERAP2, BCO2, DPY30, NPNT, GSDMB, CLEC6A, NFKBIZ, IL26, CHRFAM7A, TEX12, TRIM16	639
Duplicated Gene		131
Total Gene	676	

### One-way ANOVA

Based on the pathologic grade of patients in the TCGA-PDAC database, one-way ANOVA was used to compare differences in the expression of candidate pyroptosis and inflammasome-related genes between different stages of PDAC (Supplementary Table 4). Genes with *P* < 0.05 were considered to be the stage-related pyroptosis and inflammasome-related genes.

### Construction of the prognostic signature

To elucidate the stage-related pyroptosis and inflammasome-related genes that had significant correlations with overall survival (OS) of PDAC patients from TCGA database using univariate Cox regression analysis (*P* < 0.2). LASSO regression analysis was then applied to obtain the optimal variables for constructing a prognostic signature via ‘‘glmnet’’ package and tenfold cross-validation was utilized to ensure optimal values of the LASSO penalty parameters. A prognostic model was established on the basis of linear combinations of regression coefficient (β) and the gene expression level of the LASSO-Cox regression model (Supplementary Table 5).

### Survival analysis and receiver operating characteristic (ROC) curves

To assess the validity of the prognostic signature, TCGA-risk scores were calculated based on the expression of prognostic genes in each TCGA-PDAC sample and their corresponding coefficients, regarding the following formula:

$$risk\;score = \frac{{e^{sum\;(each\;gene^{\prime}s\;expression\;levels\; \times \;corresponding\;coefficient)} }}{{e^{sum\;(each\;gene^{\prime}s\;means\;expression\;levels\; \times \;corresponding\;coefficient)} }}$$

All TCGA-PDAC samples were divided into the high- and low-risk groups based on the median risk score (Supplementary Table 6). Survival analysis of the two groups was performed using the R software package "Survival". Survival curves were examined using the Kaplan–Meier (K–M) method to compare survival differences between risk groups. Then, time-dependent ROC curves for risk scores were created to analyze patients’ survival at 1 year, 3 years, and 5 years. To verify the generality of the prognostic signature, the above steps were repeated in the ICGC database.

### Construction of a nomogram

Independent prognostic factors were identified for TCGA-PDAC from clinical characteristics and risk score by univariate (*P* < 0.2) and multivariate Cox analysis (*P* < 0.05). A nomogram constructed based on independent prognostic factors was subsequently plotted by nomogramEx package. Correction curves were used to assess the predictive accuracy of the nomogram (combined model) for patient survival at 1, 3, and 5 years. Furthermore, decision curve analysis (DCA) curves were also performed to predict the net benefit of the combined model to the clinic.

### Functional enrichment analysis

Gene ontology (GO) enrichment analysis of candidate prognostic genes was achieved by Metascape (https://metascape.org/gp/index.html#/main/step1) and visualized in the Cytoscape package from the R software. Gene set variation analysis (GSVA) was performed in TCGA-high- and low-risk group samples using the GSVA package in R language, and the limma package was used to obtain hallmark gene sets with |t|> 3 (Supplementary Table 7), which were obtained from the Molecular Signatures Database (MSigDB; http://www.gsea-msigdb.org/gsea/msigdb/index.jsp).

### Tumor microenvironment analysis

The estimate package in R was deployed to calculate the proportion of stromal cells and immune cells in the high- and low-risk groups of TCGA (Supplementary Table 8). The proportion of immune cells infiltrating TCGA-PDAC samples (Supplementary Table 9) was explored in the GSVA package using a single sample gene set enrichment analysis (ssGSEA).

### Cytolytic activity (CYT) and tumor inflammation score

CYT was calculated using RNA-Seq data from TCGA-PDAC samples (Supplementary Table 10) based on the transcript levels of two key cytolytic effectors, granzyme A (GZMA) and perforin (PRF1), which could be utilized in this analysis to evaluate the cytotoxic immune cell activity^19^.

For the tumor inflammation scores in the high- and low-risk groups from TCGA database, which were obtained using ssGSEA. Briefly, 34 inflammation response-related factors were collected from the published reports as the inflammation gene set, and the inflammation scores of TCGA-PDAC samples were derived by the ssGSEA algorithm (Supplementary Table 11) and then compared in the high- and low-risk groups.

### Tissue samples, quantitative real-time PCR

To further validate the potential roles of signature genes in PDAC, six paired PDACs and matched normal adjacent tissue samples were collected from the SHDSYY. Ethical approval was confirmed by the ethical committee of the hospital. Tissue specimens were frozen in liquid nitrogen and stored at − 80 °C until used. Total RNA was extracted with a TRIzol Reagent (ThermoFisher: #15596018), and the concentration was calculated by the A260/A280 ratio. The PrimeScript RT reagent kit (EZBioscience: #A0010CGQ) and SYBR-Green PCR reagent (EZBioscience: #A0012-R2-L) were used to perform cDNA synthetization and further conduct RT-qPCR based on the LightCycler ® 480 System (Roche). The housekeeping gene GAPDH was used as an endogenous control. The 2−DDCT cycle threshold method was used to calculate the relative expression. Supplementary Table 3 lists the primers used in this study.

### Statistical analysis

The analyses in this study were all based on the R language. Heatmaps and Box plots were drawn using the heatmap and ggplot2 packages, respectively. A log-rank test was used for the K–M curves. The ROC curves were analyzed with the package pROC. A chi-square test was performed to determine differences in clinical characteristics between high- and low-risk groups. Unless otherwise stated, *P* < 0.05 was considered statistically significant.

### Ethics approval and consent to participate

The experimental protocol was established, according to the ethical guidelines of the Helsinki Declaration and was approved by the Human Ethics Committee of Shanghai Tenth People's Hospital. Written informed consent was obtained from individual or guardian participants.All the data used in this study was acquired from the public genomic repository whose informed consent was completed.

## Results

### Identification of candidate prognostic genes from the Stage-related pyroptosis and inflammasome-related genes in the TCGA-PDAC database

**Figure 1:**
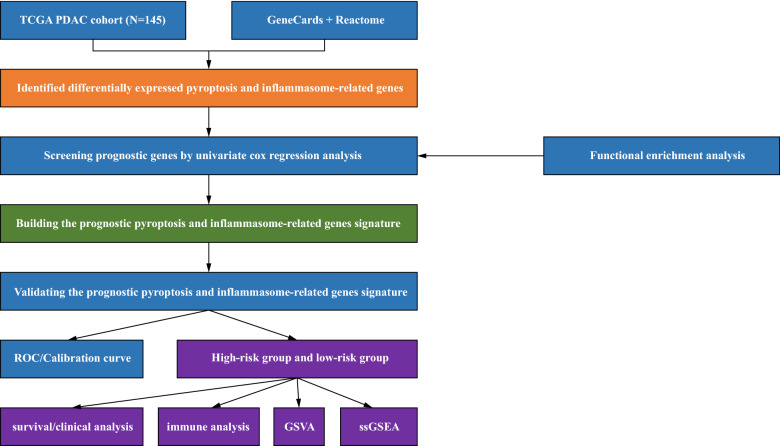
The specifc workfow graph for this study.

**Figure 2:**
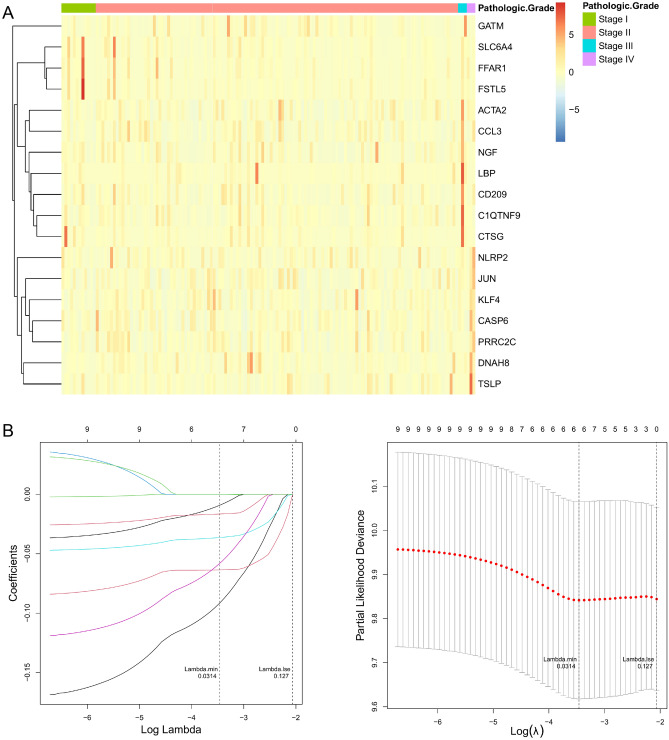
Construction of risk signature based on the expressions of the 18 pyroptosis and inflammasome-related genes. (**A**) Heatmap (green: low expression level; red: high expression level) of the pyroptosis and inflammasome-related genes between different pathologic grade in PDAC. (**B**) LASSO regression of the 9 OS-related genes and cross-validation for tuning the parameter selection in the LASSO regression.

The fowchart of data analysis was shown in Fig. 1. Upon overlapping 147 pyroptosis-related genes with 660 inflammasome-related genes, we gained a total of 676 genes defined as pyroptosis and inflammasome-related genes (Table 1). Subsequently, genes that were not expressed in more than 50% of the samples were excluded and the remaining 654 genes were used for the following analysis (Supplementary Table 3). Meanwhile, we targeted 145 PDAC samples with complete pathologic grade (Stage) information in TCGA database, which were divided into 4 subgroups, with 12 in Stage I, 127 in Stage II, 3 in Stage III, and 3 in Stage IV. Subsequently, we ascertained pyroptosis and inflammasome-related genes that were differentially expressed in each Stage subtype based on ANOVA. The Heatmap revealed that the expression of a total of 18 genes associated with pyroptosis and inflammasome had differed markedly among these subgroups (Fig. 2A). Aiming to appraise the candidate prognostic pyroptosis and inflammasome -related genes, we executed a univariate Cox analysis in R for the 18 genes mentioned above. Ultimately, we were awarded a total of nine candidate prognostic genes (*P* < 0.2), namely *ACTA2*, *C1QTNF9*, *CCL3*, *CTSG*, *DNAH8*, *GATM*, *LBP*, *NGF*, and *SLC6A4* (Table 2). The Hazard Ration (HR) values for these genes were all less than 1, which we speculated might be the protective factors for PDAC. Moreover, we revealed the potential functions of these nine genes, which were found to be closely associated with the immune response (Supplementary Fig. 1).

**Table 2:** Univariate Cox regression of 18 candidate prognostic pyroptosis and inflammasome-related genes.

Characteristics	Hazard.Ration	CI95	*P* value	HR (95% CI)
ACTA2	0.8388	0.698–1.008	0.06	0.8388 (0.698–1.008)
C1QTNF9	0.822	0.687–0.983	0.032	0.8220 (0.687–0.983)
CASP6	0.9596	0.679–1.355	0.815	0.9596 (0.679–1.355)
CCL3	0.8755	0.753–1.018	0.084	0.8755 (0.753–1.018)
CD209	0.9407	0.825–1.073	0.363	0.9407 (0.825–1.073)
CTSG	0.9112	0.823–1.009	0.073	0.9112 (0.823–1.009)
DNAH8	0.8857	0.781–1.005	0.06	0.8857 (0.781–1.005)
FFAR1	0.9665	0.888–1.052	0.432	0.9665 (0.888–1.052)
FSTL5	0.9683	0.881–1.064	0.504	0.9683 (0.881–1.064)
GATM	0.9018	0.788–1.032	0.133	0.9018 (0.788–1.032)
JUN	0.8458	0.648–1.103	0.217	0.8458 (0.648–1.103)
KLF4	1.0201	0.819–1.271	0.859	1.0201 (0.819–1.271)
LBP	0.9313	0.847–1.024	0.143	0.9313 (0.847–1.024)
NGF	0.8423	0.701–1.012	0.068	0.8423 (0.701–1.012)
NLRP2	1.0515	0.967–1.144	0.242	1.0515 (0.967–1.144)
PRRC2C	1.0008	0.765–1.309	0.995	1.0008 (0.765–1.309)
SLC6A4	0.9174	0.811–1.038	0.171	0.9174 (0.811–1.038)
TSLP	0.9891	0.826–1.185	0.905	0.9891 (0.826–1.185)

### Construction and evaluation of the pyroptosis and inflammasome-related genes based on prognostic signature

**Figure 3:**
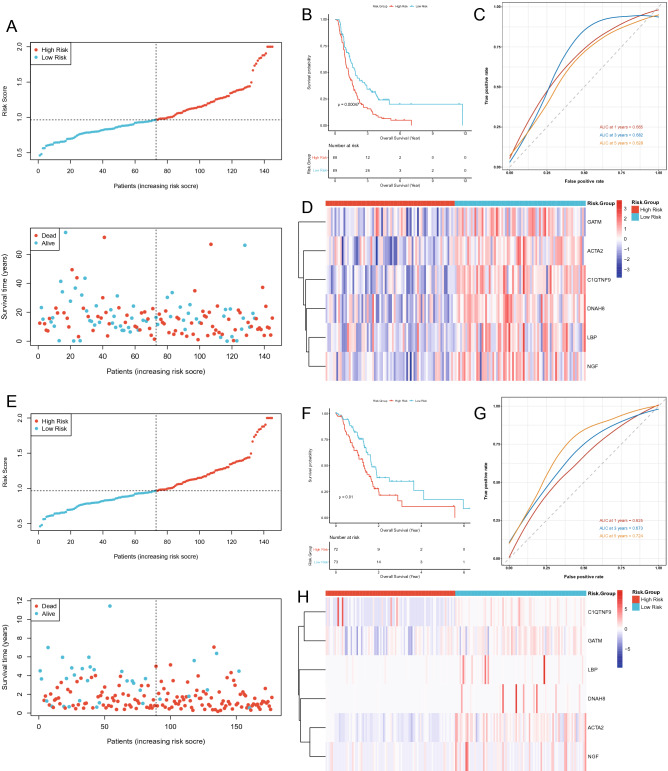
Evaluation and validation of the risk model in TCGA and ICGC cohort. (**A**) The risk curve is based on the risk score of each sample in the TCGA database (top). The scatterplot is based on the survival status of each sample (bottom). The bright and red dots represent low-risk group/survival and high-risk group/death, respectively. (**B**) Kaplan–Meier curves for comparison of the OS between low- and high-risk groups. (**C**) ROC curves demonstrated the predictive efficiency of the risk score. (**D**) Heatmap demonstrating the distribution of the six pyroptosis and inflammasome-related gene expressions in the TCGA cohort. (**E**) The risk curve is based on the risk score of each sample in the ICGC database (top). The scatterplot is based on the survival status of each sample (bottom). The bright and red dots represent low-risk group/survival and high-risk group/death, respectively. (**F**) Kaplan–Meier curves for comparison of the OS between low- and high-risk groups. (**G**) Time-dependent ROC curves for PDACs. (**H**) The heatmap displayed the expression levels of pyroptosis and inflammasome-related genes in the high-risk and low-risk groups.

A descending analysis of the 9 candidate prognostic genes was further pursued in TCGA database by LASSO regression analysis to retrieve the optimized variables for generating a prognostic signature. Ultimately, we constructed a 6-gene prognostic signature based on *ACTA2*, *C1QTNF9*, *DNAH8*, *GATM*, *LBP*, and *NGF* (Fig. 2B). Risk scores were calculated for each patient in the TCGA according to the previous formula and patients were categorized into the high- (n = 72) and low-risk (n = 73) groups based on the median risk score. The scatter plot suggested that as the risk score of the sample climbed, the group of patients who died became progressively larger (Fig. 3A). In the TCGA database, patients in the high-risk group appeared to face the inferior OS (*P* = 0.01; Fig. 3B). Subsequently, the ROC curve for assessing the predictive strength of the 6-gene prognostic signature exhibited an AUC of 0.665, 0.682, and 0.628 at 1, 3, and 5 years, respectively (Fig. 3C), suggesting that the risk signature would have a tolerable predictive capacity in the TCGA database. Furthermore, based on the Heatmap of expression patterns for the 6 prognostic genes between the two risk groups, an overexpression of all genes in the low-risk group with a better OS was evident (Fig. 3D), suggesting that the high expression of these genes in patients might be an indicator of a good outcome, which also matched our previous inference. Furthermore, the detailed statistical table for clinical information was displayed in Supplementary Table 12 (Supplementary Fig. 2).

### Validation of the 6 prognostic gene signatures in the ICGC database

Herewith, we would execute the same analysis to demonstrate the general applicability of the 6-gene based prognostic signature in the external validation set, which was derived from the ICGC database and contained 177 PDAC samples with complete clinical information. Based on the expression of the six prognostic genes in the ICGC database, we recalculated the risk score for each ICGC-PDAC sample. Again, based on the median value of the risk score, 88 samples were included in the high-risk group, while the remaining 89 samples were categorized in the low-risk group (Supplementary Table 13). Similar to the previous results in TCGA database, there was an aggregation of deceased cases in the high-risk group (Fig. 3E). Likewise, the K–M curves presented a worse OS for patients in the ICGC-high-risk group compared to the ICGC-low-risk group (*P* = 0.00047; Fig. 3F). Concurrently, this signature displayed similar results for ICGC-PDAC in ROC curve analysis at 1, 3, and 5 years, with AUCs of 0.625, 0.673, and 0.724, respectively (Fig. 3G). Although *C1QTNF9* was overexpressed in very few samples from the high-risk group, when considered together, the expression patterns of the six prognostic genes in the high- and low-risk groups in the ICGC database were consistent with those in the TCGA database (Fig. 3H). The above results suggested that the prognostic signature based on the 6 genes has a stable and generally applicable predictive validity.

### The risk score and PORT were the independent prognostic elements for TCGA-PDAC patients

**Figure 4:**
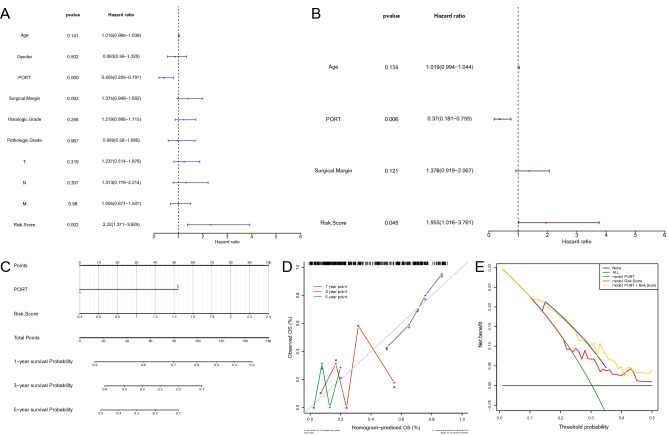
Construction of a predictive nomogram. (**A**, **B**) Hazard ratio and *P* value of the constituents involved in univariate and multivariate Cox regression considering clinical parameters and Risk Score in PDAC. (**C**) Nomogram to predict the 1-year, 3-year, and 5-year overall survival rate of PDAC patients. (**D**) Calibration curve for the overall survival nomogram model in the discovery group. A dashed diagonal line represents the ideal nomogram. (**E**) DCA curve was established to evaluate the clinical utility and benefit of the nomogram and additional parameters.

From the risk score and numerous clinical characteristics, a univariate Cox regression analysis pointed to Age, PORT, Surgical Margin, and risk score as the elements associated with independent prognosis in PDAC (*P* < 0.2; Fig. 4A). Multifactorial Cox analysis ultimately identified two independent prognostic factors, risk score and PORT (Fig. 4B). Subsequently, a Nomogram was constructed based on those 2 factors to provide a quantitative method for predicting the likelihood of 1-year, 3-year, and 5-year OS in PDAC patients (Fig. 4C). The total points were passed through the sum of the corresponding scores for each patient's status paired with the corresponding factor, where a higher total point for the patient represented a worse outcome. The calibration curves suggested that the predictive performance of the combined model (Nomogram) for patient OS in 3- and 5-year was probably overestimated, using the ideal situation as a reference, but the predictiveness for 1-year OS was more reliable (Fig. 4D). The DCA demonstrated that the combination model exhibited the optimal net benefit for 1 year OS (Fig. 4E). Unfortunately, due to the limitation of the sample size (8 for OS greater than or equal to 3 years), we were unable to predict the net benefit of the combined model for 3- and 5-year OS.

### Biological differences between the high- and low-risk groups

**Figure 5:**
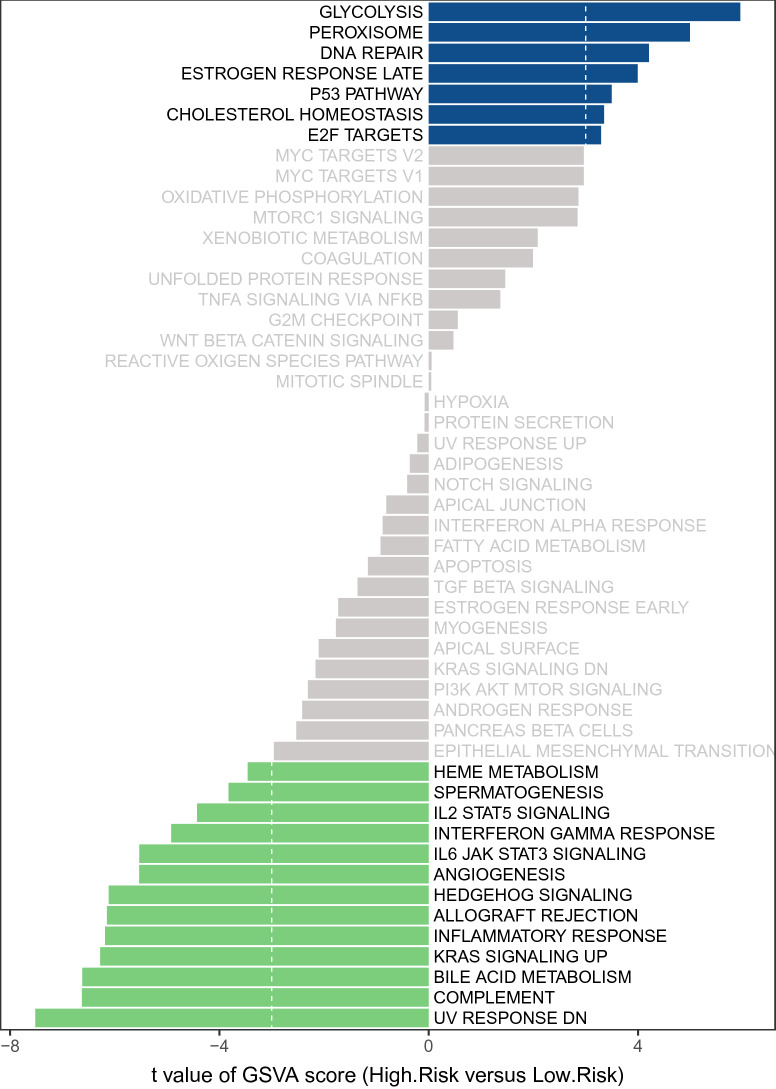
A gene set variation analysis with the hallmark gene set. Differences in pathway activities scored by GSVA between high- and low-risk patients. T values are shown from a linear model. We set |t|> 3 as a cutoff value. The midnight blue column indicates activated hallmark gene sets in high-risk patients, and the green column indicates activated hallmark gene sets in low-risk patients.

To further reveal the underlying mechanisms of OS differences between the high- and low-risk groups, we performed a GSVA with the hallmark gene set as a pre-determined gene set (Fig. 5). The terms that were significantly activated in the high-risk group compared to the low-risk group were ‘GLYCOLYSIS’, ‘PEROXISOME’, ‘DNA REPAIR’, ‘ESTROGEN RESPONSE LATE’, ‘P53 PATHWAY’, ‘CHOLESTEROL’, ‘HOMEOSTASIS’, and ‘E2F TARGETS’ (|t| ≥ 3). Notably, several studies have demonstrated that activation of the glycolysis pathway facilitates the malignant progression^20^ and poor prognosis^21^ of PDAC. Meanwhile, oncogenic mutations and dysregulation of P53 lead to changes in pancreatic cell metabolism thus driving PDAC^22^. Moreover, an imbalance in cholesterol homeostasis induced by a high-fat diet has also been suggested as a risk factor for PDAC^23^. Therefore, we hypothesized that the activation of these terms directly or indirectly influenced the poorer OS of patients in the high-risk group. The low-risk group was mainly associated with signaling pathways such as ‘UV RESPONSE DN’, ‘COMPLEMENT’, and ‘BILE ACID METABOLISM’. However, we noted that the ‘KRAS SIGNALING UP’ pathway was significantly different in the two groups. Keep in mind that numerous molecular studies have shown that KRAS mutations are the initiating genetic event in PDAC^24,25^. Further, the enrichment of ‘INFLAMMATORY RESPONSE’ and inflammatory signaling (‘IL6 JAK STAT3 SIGNALING’ and ‘IL2 STAT5 SIGNALING’) pathways in the low-risk group might imply a higher degree of infiltration of anti-cancer immune cells (e.g., CD8^+^ T cells and dendritic cells) in this group of patients^26^. Moreover, the difference in the ‘ANGIOGENESIS’ pathway between the two groups was supported by the concept that vascular density was positively associated with PDAC progression^27^. Above, GSVA partially illustrated the biological differences between the high- and low-risk groups at the pathway level.

### Immune landscape analysis of PDAC

**Figure 6:**
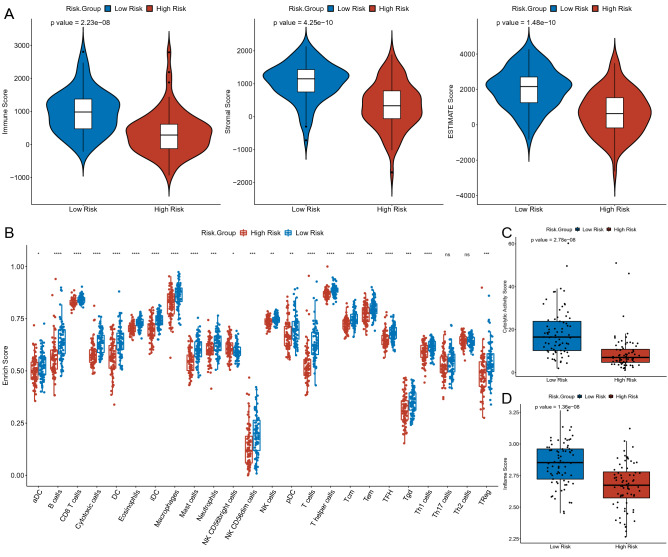
Biological differences between the high- and low-risk groups. (**A**) The violin plot showed the difference in Immune Score (left), Stromal Score (middle), and ESTIMATE Score (right) between high- and low-risk groups. (**B**) Comparison of the enrichment scores of 24 types of immune cells between low- (green box**)** and high-risk (red box**)** groups in the TCGA cohort. (**C**) Boxplots show the distribution of CYT score (top) and Inflame score (bottom**)** in the low-risk group versus the high-risk group.

Inspired by the results of this study and previous studies^28^, we turned our attention to the effect of risk scores on the immune microenvironment of PDAC patients. The ESTIMATE algorithm suggested that the immune, stromal, and ESTIMATE scores were significantly higher in the low-risk group versus the high-risk group (Fig. 6A). The ssGSEA then imputed the abundance of immune infiltrating cells between the high- and low-risk groups. The results suggested that the abundance of 22 of the 24 immune infiltrating cells was significantly different between the two groups. Exhaustively, in addition to NK CD56bright cells, aDC, B cells, CD8 T cells, cytotoxic cells, DC, eosinophils, iDC, macrophages, mast cells, neutrophils, NK CD56dim cells, NK cells, pDC, T cells, T helper cells, Tcm, Tem, TFH, Tgd, Th1 cells, and TReg were all found to have a high enrichment score in the low-risk group (Fig. 6B). Subsequently, with the average expression of GZMA and PRF1, we examined the immune cell-mediated CYT. The results showed that the CYT scores were significantly higher in the low-risk group of patients with longer OS than in the high-risk group (Fig. 6C). Interestingly, which was in agreement with previous studies, higher CYT was positively associated with prolonged survival time in a variety of cancers (e.g., colorectal and pancreatic cancers)^29,30^. Besides, we calculated the inflammatory characteristic scores of the tumors and found that the low-risk group exhibited higher inflammation scores compared to the high-risk group (Fig. 6D).

### Tissue samples, quantitative real-time PCR

**Figure 7:**
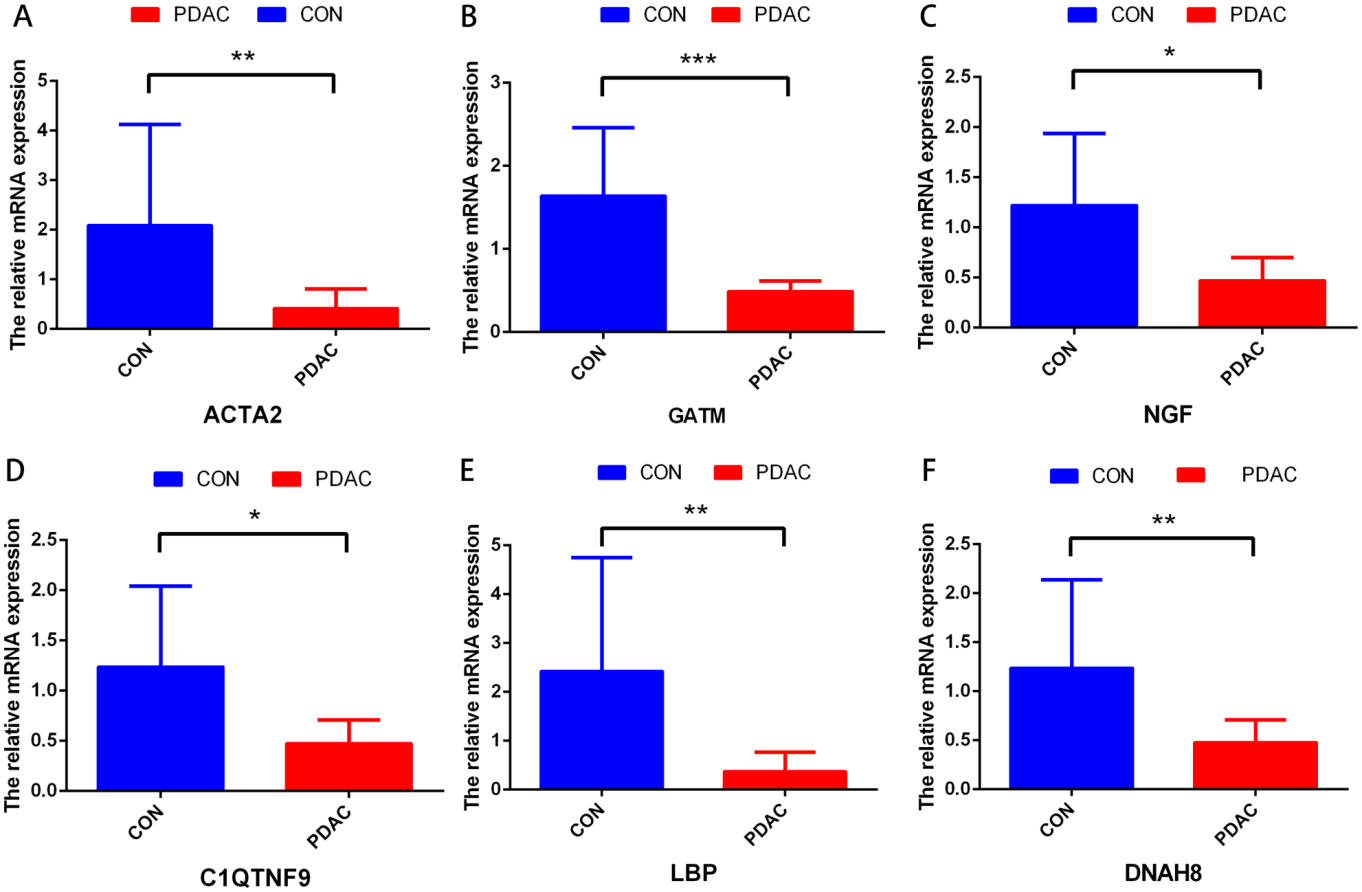
The expressions of six signature genes were validated by quantitative real-time PCR (qRT-PCR). (**A**–**E**) Expression of genes at the mRNA level by qRT-PCR. qRT-PCR, quantitative real-time PCR. *P < 0.05; **P < 0.01; ***P < 0.001. ns, no significance.

To verify the expression level of signature genes in PDAC, we collected six paired cancer- and adjacent normal tissues from SHDYSS. As shown in Fig. 7A–F, qRT-PCR showed that the expression of the ACTA2, C1QTNF9, DNAH8, GATM, LBP, and NGF were significantly downregulated in tumor samples. Briefly, it was downregulated in six patients.

## Discussion

Pancreatic ductal adenocarcinoma (PDAC) is the most prevalent and aggressive type of pancreatic cancer^31,32^. Despite great efforts in developing novel therapies and technologies for PDAC, the overall mortality and morbidity rates have increased in recent years and are expected to increase further in the future^33^. Therefore, effective prognostic biomarkers are urgently needed for PDAC. In the present study, a novel prognostic model of PDAC based on 6 pyroptosis and inflammasome-related genes was constructed, which effectively evaluated the survival and immune microenvironment of patients.

Pyroptosis is a form of inflammatory cell death and is mainly regulated by inflammasome-related pathways^34^. On one hand, normal cells are stimulated by a large number of inflammatory factors released by pyroptosis, leading to their transformation into tumor cells^35^. On the other hand, targeting pyroptosis in tumor cells may be a new therapeutic strategy^36^. In PDAC, how pyroptosis and inflammasome-related genes interact and whether they are related to patient survival time remains unknown. In this study, we first studied 676 currently known pyroptosis and inflammasome-related genes and ascertained 18 genes of them that were associated with pathologic grade. A 6-gene risk signature was further constructed according to prognostic value via univariate Cox and LASSO regression analysis. Based on the 6-gene risk signature, the risk scores were calculated in TCGA database and proven of a valuable prognostic sense in ICGC database. Additionally, GSVA analysis illustrated the biological differences between the high- and low-risk groups at the pathway level. Moreover, the high-risk group had universally reductive levels of infiltrating immune cells as well as cytolytic and inflame activity compared with the low-risk group.

The 6 pyroptosis and inflammasome-related genes (*ACTA2*, *C1QTNF9*, *DNAH8*, *GATM*, *LBP*, and *NGF*) were generated and could predict survival in PDAC patients. Actin alpha 2 (*ACTA2*), also known as a-SMA, is initially identified to function in cell-generated mechanical tension as well as maintenance of cell shape and movement^37^. Subsequent researches confirmed that the dynamics of cytoskeletal structures affected by ACTA2 could be pivotal to metastasis in lung adenocarcinoma^37,38^. In addition, ACTA2 was currently considered to be a marker of the epithelial-mesenchymal transition (EMT) process of tumors. Recently, ACTA2 was known as an indicator of pyroptosis-induced myofibroblast activation and an inducer to activate the inflammasome^39,40^. Nevertheless, the role of ACTA2 in PDAC remains unclear. In the present study, ACTA2 might promote the progression of PDAC, as it was upregulated significantly in the low-risk group, which provides some insight for further studies. C1q and TNF-related 9 (*C1QTNF9*) were indicated to attenuate atherosclerosis through the AMPK-NLRP3 inflammasome singling pathway and were frequently reported in the cardiovascular system^41,42^. Rarely have studies reported an association between C1QTNF9 and cancer. In our study, the expression of C1QTNF9 in low-risk patients is increased, which might exert a crucial effect on the prognosis of PDAC. Researches on dynein axonemal heavy chain 8 (*DNAH8*) was mainly focused on abnormalities of sperm and male infertility^43,44^. In cancer, DNAH8 was proposed to be associated with the prognosis of prostate cancer and hepatocellular carcinoma^45,46^. Glycine amidinotransferase (*GATM*) in mitochondria was associated with increased ROS production, activation of the NLRP3 inflammasome, enhanced secretion of the profibrotic cytokine IL-18, and increased cell death^47^. GATM as the rate-limiting enzyme for creatine synthesis enhances cancer metastasis and shortens mouse survival by upregulation of Snail and Slug expression^48^. Moreover, GATM in adipocytes was proved to be required for obesity-driven tumor progression^49^. In this study, low expression of GATM predicted poor survival rates, indicating that it functioned as a tumor suppressor in PDAC. The lipopolysaccharide-binding protein (LBP) is critically involved in innate immune responses. LBP serves not only as an extracellular lipopolysaccharide (LPS) shuttle but in addition, facilitates intracellular transport of LPS, which activates macrophage into M1 type and induces a highly inflammatory type of pyroptosis^50^. However, the specific mechanisms by which LBP reduces the survival rate of PDAC patients still need further exploration. Nerve growth factor (NGF) in acquired immune responses. In human monocytes and null THP-1 cell line, NGF significantly upregulates IL-1β in a caspase-1 dependent manner through NLRP1/NLRP3 inflammasomes^51^. In summary, 2 genes (ACTA2 and LPB) in the prognostic model were proven to promote pyroptosis, and 4 genes (C1QTNF9, DNAH8, GATM, and NGF) were identified to be associated with inflammasome pathway. Nevertheless, how these genes interact with each other in PCDA patients remains to be further investigated.

The key anti-tumor infiltrating immune cells, especially DC, Cytotoxic cells, and NK cells, have lower levels in the high-risk group, indicating an overall impairment of immune functions. However, regulatory T cells (Tregs), traditionally recognized as immunosuppressive cells and correlated with poor prognosis^52^, but were risen in the low-risk group in this study. One possible reason for this complication might be that Treg cells are essential for regulating the overactive inflammatory response caused by the activation of pyroptosis and inflammasomes pathway in the tumor microenvironment. Additionally, the levels of cytolytic and inflame activity were increased in the low-risk group. Therefore, the pyroptosis and inflammasome-related genes defined in this study could predict the immune microenvironment of PDAC, which might guide immunotherapy in the future.

Meanwhile, there are some limitations in our study. Firstly, the clinical information downloaded from the TCGA is incomplete, especially the therapy, which may be helpful to understand whether pyroptosis and inflammasome-related genes are biomarkers of treatment. Secondly, the mechanism how pyroptosis and inflammasome modulate the precise process of PDAC is unclear. Lastly, the prognostic model needs to be verified in a large-scale and multicenter clinical cohort.

Collectively, the present study raised a brand-new prognostic model for PDAC patients based on pyroptosis and inflammasome-related genes. Mechanically, the heterogeneity of biology and alteration of the immune environment within our model had been demonstrated, which deepens the molecular understanding and might direct the therapy strategy of PDAC.

## Conclusions

Our study revealed the prognostic role of the pyroptosis and inflammasome-related genes in PDAC. Simultaneously, the biological and prognostic heterogeneity of PDAC had been demonstrated, deepening our molecular understanding of this tumor.

## Supplementary Information

Supplementary Information 1.

Supplementary Information 2.

## Data Availability

All data used in this study were available in The Cancer Genome Atlas (TCGA) [https://portal.gdc.cancer.gov/] and International Cancer Genome Consortium (ICGC) [https://dcc.icgc.org/] repository.

## Abbreviations

PDAC: Pancreatic ductal adenocarcinoma
ANOVA: Analysis of variance
LASSO: Least absolute shrinkage and selection operator
GSVA: Gene set variation analysis
TCGA: The Cancer Genome Atlas
ICGC: International Cancer Genome Consortium
ACTA2: Actin alpha 2
C1QTNF9: C1q and TNF related 9
DNAH8: Dynein axonemal heavy chain 8
GATM: Glycine amidinotransferase
LBP: Lipopolysaccharide binding protein
NGF: Nerve growth factor
IL-1b: Interleukin-1b
IL-18: Interleukin-18
NLRP1: NLR family pyrin domain containing 1
NLRP3: NLR family pyrin domain containing 3
NLRC4: NLR family pyrin domain containing 4
AIM2: Absent in melanoma 2
GSDMD: Gasdermin D
GSDMD-NT: Gasdermin D N-terminal
NSCLC: Nonsmall-cell lung cancer
MST1: Mammalian STE20-like kinase 1
OS: Overall survival
ROC: Receiver operating characteristic
K–M: Kaplan–Meier
GO: Gene ontology
MSigDB: Molecular signatures database
ssGSEA: Single sample gene set enrichment analysis
CYT: Cytolytic activity
GZMA: Granzyme A
PRF1: Perforin 1
CCL3: C-C motif chemokine ligand 3
CTSG: Cathepsin G
SLC6A4: Solute carrier family 6 member 4
HR: Hazard ration
PORT: Post-operative radiotherapy
LPS: Lipopolysaccharide
Tregs: Regulatory T cells
