# Supplementary material for: A novel refined pyroptosis and inflammasome-related genes signature for predicting prognosis and immune microenvironment in pancreatic ductal adenocarcinoma
Source: Sci Rep. 2022 Nov 1;12:18384. doi: 10.1038/s41598-022-22864-z (PMC9626462; doi:10.1038/s41598-022-22864-z)
Supplement: Supplementary file 1 — Supplementary Information 1. [file 41598_2022_22864_MOESM1_ESM.docx]

Supplementary Figures


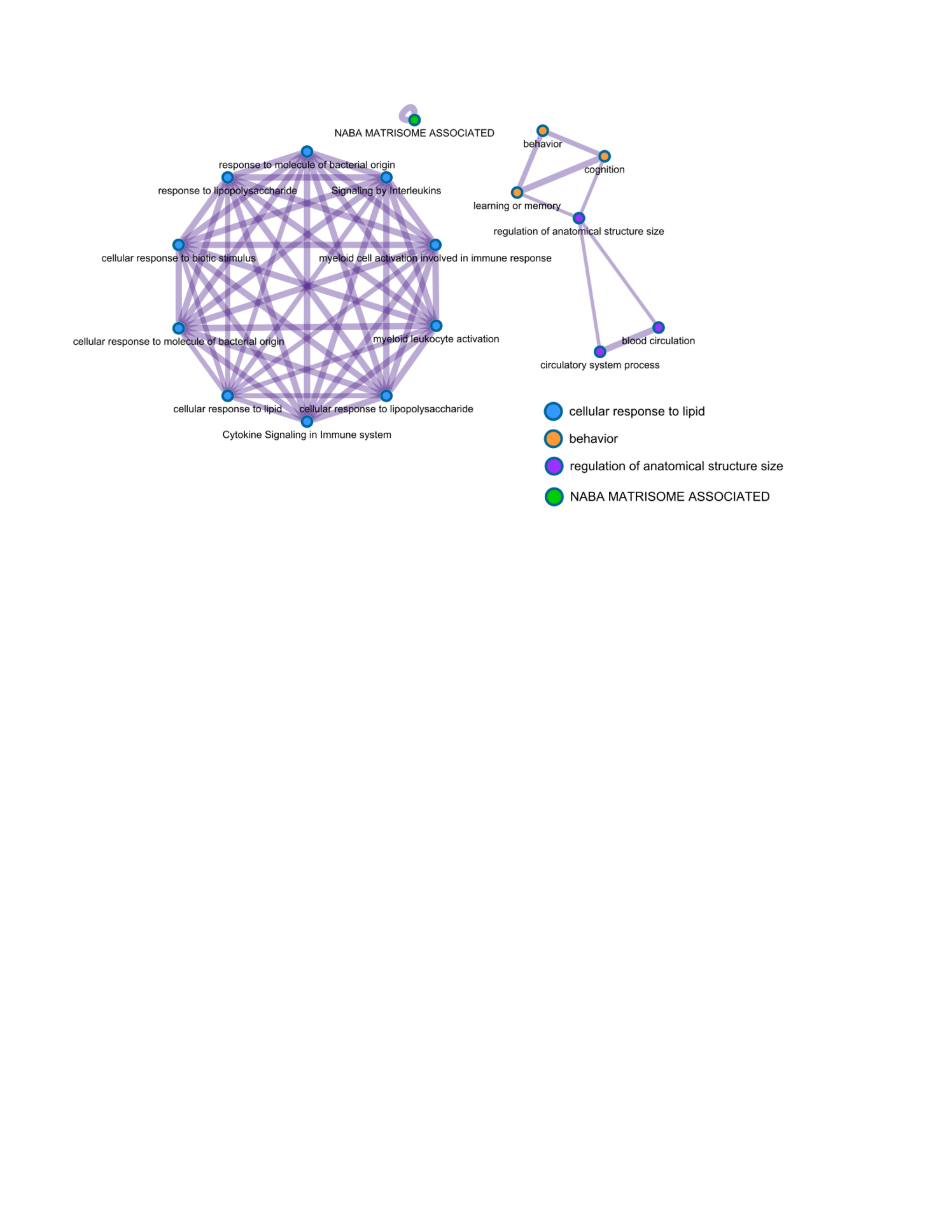


**Supplementary Figure 1.** **The enrichment network of representative terms is performed with Cytoscape.** Each term is represented by a circle node, the size of which is proportional to the number of input genes falling into that term, and the color represents its cluster identity. One term from each cluster is selected to have its term description shown as label.


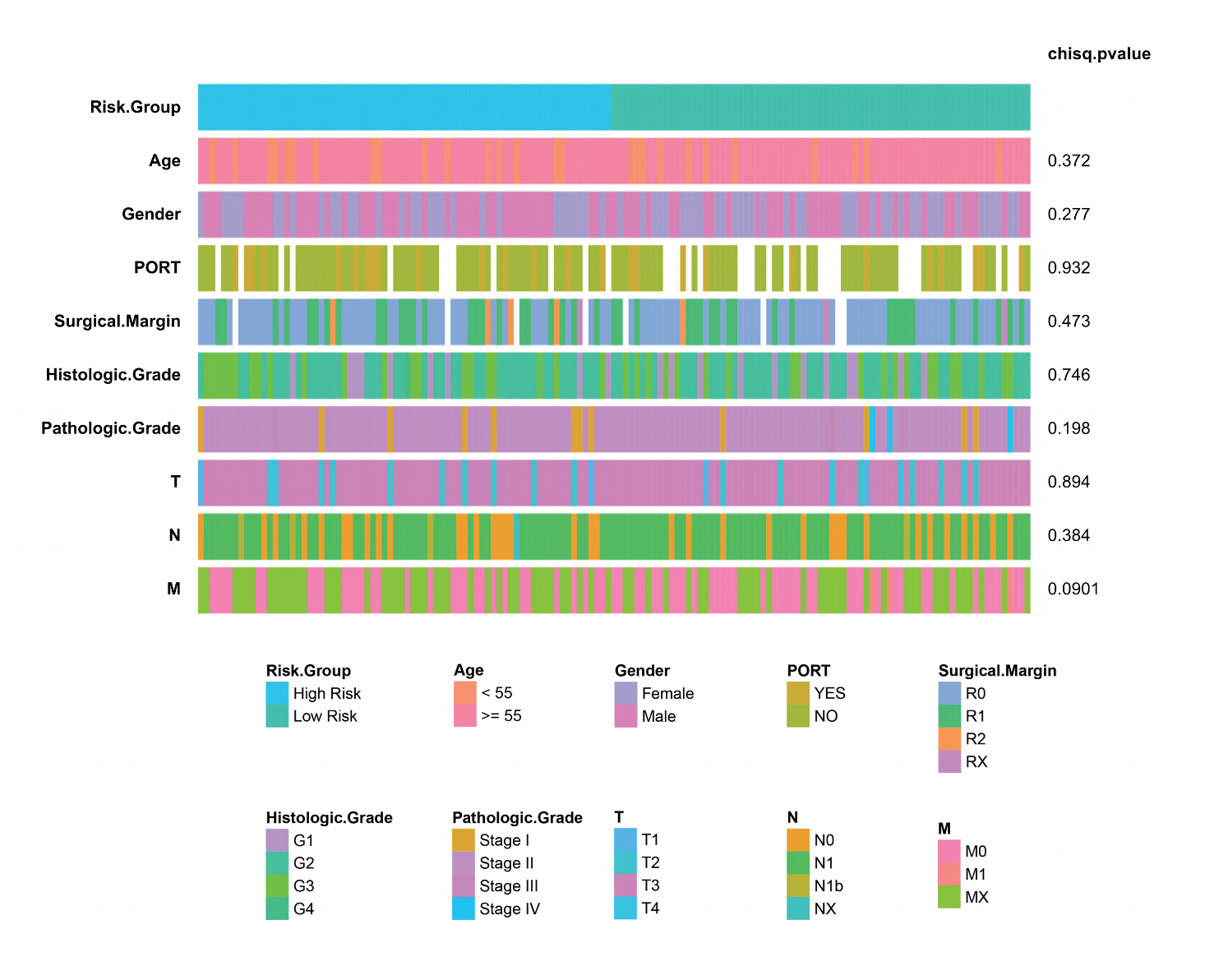
 **Supplementary Figure 2.** **Heatmap for the connections between clinicopathologic features and the risk groups.**
